# Leveraging deep learning and big data to enhance computing curriculum for industry-relevant skills: A Norwegian case study

**DOI:** 10.1016/j.heliyon.2023.e15407

**Published:** 2023-04-11

**Authors:** Muhammad Umair Hassan, Saleh Alaliyat, Raheem Sarwar, Raheel Nawaz, Ibrahim A. Hameed

**Affiliations:** a Department of ICT and Natural Sciences, Norwegian University of Science and Technology (NTNU), Ålesund, Norway; b Department of Operations, Technology, Events and Hospitality Management, Manchester Metropolitan University, Manchester, United Kingdom; c Staffordshire University, Staffordshire, United Kingdom

**Keywords:** Computing education research, Curriculum development, Internet education, Deep learning, Student experiences, Employability

## Abstract

Computer science graduates face a massive gap between industry-relevant skills and those learned at school. Industry practitioners often counter a huge challenge when moving from academics to industry, requiring a completely different set of skills and knowledge. It is essential to fill the gap between the industry's required skills and those taught at varsities. In this study, we leverage deep learning and big data to propose a framework that maps the required skills with those acquired by computing graduates. Based on the mapping, we recommend enhancing the computing curriculum to match the industry-relevant skills. Our proposed framework consists of four layers: data, embedding, mapping, and a curriculum enhancement layer. Based on the recommendations from the mapping module, we made revisions and modifications to the computing curricula. Finally, we perform a case study of the Norwegian IT jobs market, where we make recommendations for data science and software engineering-related jobs. We argue that by using our proposed methodology and analysis, a significant enhancement in the computing curriculum is possible to help increase employability, student satisfaction, and smart decision-making.

## 1 Introduction

The primary objective of Computing Education Research (CER) is to improve the computing educational framework through the development of a comprehensive framework to promote high-quality computing research, nurture the learning process, and identify feasible routes for how computing is taught and learned [1]. In addition to enhancing innovation and research potential, the development of students' computing skills and capacities would suggest a transition toward digital transformation and sustainable innovation [2,3]. CER is regarded as a reliable indicator of students' professional development [4] in computing. It is related to significant technological advances in multiple fields, including oil & gas [5], agriculture [6], and industry [7], etc. Technological development is widely witnessed within engineering domains, e.g., computer science (CS), mathematics, electrical engineering, etc., and it is clearly seen that a significant investment in the technology sector ultimately results in better economic growth for countries [8] and also it is necessary for bringing the sustainability.

Norway is promoting research and development in various strategic sectors such as shipping, marine science, fisheries, aquaculture, and oil & gas over the past decade [9]. These industries are digitizing their operations. However, there is a lack adequate research planning for the capacity-building that will enhance the skills of Norwegian youth to face the technological change on the Norwegian labor market. In addition, industries desire to recruit candidates with higher-order cognitive abilities from the information technology (IT) sector who possess the necessary skill set [10,11]. There is a significant disparity between the industries' required skills and the acquired skills for those positions.

It is the responsibility of universities to provide recent graduates with the knowledge and skills they need to start working. In data science and software engineering, this entails imparting soft skills in addition to the fundamental theoretical knowledge required by the sector in computer science and related fields [12]. The industry's data-driven analysis of required skills is vital to fill the void for varsity graduates. Some studies related to this have been proposed, such as Tenhunen et al. [12] presented a study to produce industry-ready graduates. Their study is based o interviews, and no new knowledge about the technical development of the framework is produced in their research. Ramakrishnan et al. [13] presented a case study for achieving industry-aligned skills using a conceptual framework of industry practical digital commons (IPDC). They created a digital commons to operate as a link between academic curriculum and new business practices. Knowledge is generated and shared online in a digital commons. Their case study explains the inspiration for an IPDC and how it was created, developed, and evaluated to help students gain knowledge that applies to the workplace. They evaluated their study at an Australian university. One of the limitations of their study is that their model lacks receiving feedback over extended time periods. However, we aim to develop a technical solution that can incrementally be updated with the data from online *Job* search engines and provide relevant feedback to industrial practitioners and academia.

In this study, we argue that aligning the skills of the young Norwegian population in higher education institutions with the pertinent technology employment market and analyzing the disparity between the market and university curriculum [14] are of the utmost importance. This type of analysis can increase students' awareness of study planning tools [[15], [16], [17]], aid in the development of their career path [18,19], and improve their engagement and retention [20]. No study has, to our knowledge, presented a data-driven gap analysis between “required skills” on the Norwegian IT employment market and “acquired skills” of university students from their training and education. Our proposed model made fivefold contributions to course planners and learners.

• Firstly, we make use of the big job data presented on the web about IT-related jobs in the Norwegian job market.
• Secondly, by defining the necessary competences and abilities for Norway's digital transformation, we study the most recent trends for the skills needed to promote the social effect of learning.
• Thirdly, we analyze the significance of market's required skills with the students' required skills and try to map the gap for facilitating the data integration from several sources by employing case-based reasoning (CBR).
• Fourthly, by determining the discrepancy between the “required” and “acquired” abilities in the market, we offer a curriculum enhacement module based on the necessary skills for computer science students.
• Finally, to advance the strategic goals of Sustainable Digital Transformation (SDT) through developing Norway's sustainable policies, we aim to deliver innovative research with our proposed work by developing a quad-layered computing curriculum enhancement framework to provide fresh insights on the latest IT-jobs trends in Norwegian IT market.

Our proposed framework consists of data, word embedding, mapping, and a curriculum enhancement layer. The data layer takes two types of input data: (i) candidates' resumes and (ii) job listings from online job portals. We apply the text-based object detection [21] to detect and segment the relevant text from the resumes and job portals. Data cleansing and preprocessing are applied in the data layer, and then we shift the preprocessed data to the word embedding layer. FastText [22] is applied to extract keywords from the preprocessed data. In the third layer, we apply CBR to map the required skills with the candidate's acquired skills. Finally, we apply a curriculum enhancement layer to update the computing curriculum with market-relevant skills.

To perform this study, we aim to answer a few research questions.

• **RQ1:** How do we find required IT market skills from the industry for computing students in higher education?
• **RQ2:** How to make required IT market skills available to academic teachers and learners?

**Fig. 1:**
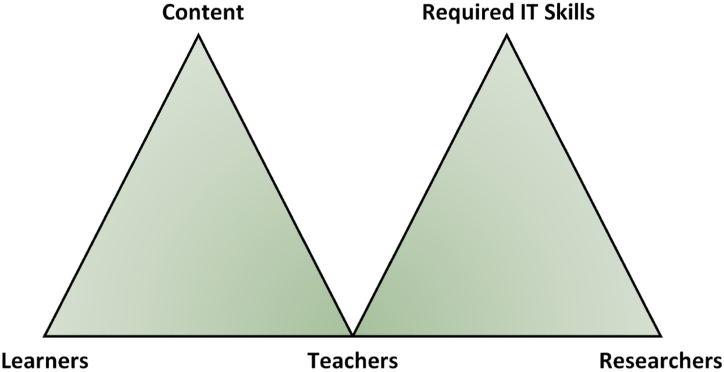
Herbst's didactic triangle [23] modified for this proposed study.

We leverage upon Herbst's didactic triangle [23] by modifying it [1] according to the prerequisites of this proposed study, where we first investigate the required skills from the industry and map it with acquired skills with the help of researchers from CER and then make it available in student's curricula. Fig. 1 is the illustration of our modified didactic triangle. A recent work by Wensheng Wu [24] investigated the internship experiences of data science students to enhance the computing curriculum according to the industrial needs by performing a survey consisting of several questions, and the main participants were the students. We, in this work, design a case study where, by collecting, the resumes from several institutes in Norway, we try to map the industry's required skills with industry's acquired skills and present a framework that can be used in academia to enhance the computing student's capacity to learn industry-relevant skills.

This research directly contributes to AI and machine learning by aligning Norway's technology market demands to the complex IT job, skill, and competence profiles associated with SDT objectives [25], which are based on the UN's sustainable development goals (SDGs). Second, by enhancing the computing curriculum for long-term innovation and digital transformation, we offer the Norwegian IT industry and academic institutions a cost-effective and efficient way to meet industrial demands. This study links state-of-the-art research and development skills to the expected IT labor market and labor needs, organizing a scientific, end-to-market strategy for Norwegian academics and industry.

The organization of this paper is as follows. Section 2 presents the relevant literature of this study. The methodological framework is briefly explained in Section 3. A case study on the Norwegian IT jobs market is available in Section 4. We have provided a comprehensive discussion on the study in Section 5 while Section 6 highlights the internal and external threats of validity. Finally, we conclude and make future work recommendations in Section 7.

## 2 Literature review

This section is organized based on the three key indicators that make the basic foundations of our proposed study.

### 2.1 Computing Education Research

Computing education is one of the key strategic areas in STEM education that is bringing revolution throughout the world [26,27]. The primary goal of CER is to discover and design better computing learning processes. Nelson et al. [28] argue that a theory-driven model can accelerate computer science learning. They propose that *theory* can describe a design, structure the design space, and help generate new designs for learning processes. A case study intervening the education design and student's study behavior was conducted by Lorås and Aalberg [29], in which they tried to map the student's engagement in organizations, independent study environments, students planning and work priorities, and time engagement. Their analysis confirms that the study design process closely relates to students' study environment. The analysis can also help computing education teachers adjust education design parameters that can help increase the student's tendency towards computing learning in academic environments.

Szabo et al. [30] presented a bibliographic study on learning theory citations in CER. In that, they provided a taxonomy for classifying the learning theory relationship strengths. The analysis was performed on 84 learning theories. The authors in Ref. [31] investigated a pilot study that assesses the use of online collaborative, integrated development environments (IDEs) in a first-year computer science classroom. Virtual computer laboratories were employed in the research to simulate some of the operations carried out in virtual computing labs. While the students worked remotely on programming, a tutor offered real-time assistance and comments. The report examines the benefits and drawbacks of the two employed platforms. The future use of virtual computer laboratories was indicated as being desirable by both the students and the instructors.

As this study also focuses on the education of Data Science students, in this regard, Sebastain Krings [32] produced an experience report concentrating on a student-organized curriculum, involving a maximum of the students’ attention where the students not only learned efficiently but they also became co-producers of the knowledge.

### 2.2 Employability based on industry-relevant skills

The term *employability* can be related to both those seeking the job and on the job. The industry seeks employees who have sufficient skills to progress in their daily routine work. A definition by the UK government [33] refers to employability as the development of personal skills that enable individuals to develop skills, knowledge, and technology, enabling them to enter and remain in employment throughout their work-life. Tenhunen et al. [12] explored the issues of preparing software engineering students for the industry and the gaps between what they learn at university and what is expected of them in the workplace. The research compares the Software Development Academy (SDA), an internal software startup, to typical capstone projects in order to suggest a fresh method for teaching industry-relevant skills. To determine the educational characteristics of the SDA program, the authors conducted 15 semi-structured interviews with SDA graduates. The most significant features of SDA, which gave students a broad skill set for their future employment, were found to be working with production-quality software and having a wide variety of responsibilities.

Kalra et al. [10] indicated that it is essential for computing graduates to develop industry-relevant skills. Employers often complain that CS graduates lack soft and higher-order thinking levels. Therefore institutes need to develop those industry-relevant skills in students by updating their curriculum rather than using traditional methods. Ruthotto et al. [11] performed a study on the IT labor market where they assessed the role of online graduate students in the job market. Their statistics show that online education for STEM disciplines has become an essential tool, and further studies are required to propose efficient learning environments for students. Ismini Vasileiou [34] claimed that with the rise of the digital transformation age, it is crucial to implement industryaccepted standards and a rigorous mapping between institutes and industrial employers to provide a holistic learning experience to cyber-security graduates. DeMark and Kozyrev [35] state that individuals seek for the shortest paths to grab job opportunities, and employers also search for the shortest routes to hire the skilled workforce. They mapped the skills with those of industrial requirements. It is beneficial for students to develop those competencies that align their skills with career opportunities. Furthermore, it is valuable for institutes to map the skills, enabling them to provide pathways to students. The employers also benefit from the skills mapping and help them gain access to the talent that has been under-identified due to several factors. Nobari et al. [17] used a community question-answering method to produce an approach that bridges the skill gap between the industry and the candidates. Their method is based on the Voteshare scoring approach, which efficiently overcomes the vocabulary gap issues.

Simmons et al. [36] indicated the gaps in the CS curriculum by devising an exploratory study. Their study explored that the current technology industry needs to make satisfactory recommendations to enhance the CS curriculum by updating the technical skills and soft skills that are essential for CS graduates to excel in their careers. Rowe et al. [37] propose that work-integrated learning is a key measure to promote students’ employability. It is based on several factors such as networking, professional skills, active project participation, etc. They indicate that enhancing the curriculum design results in the promotion of employability.

### 2.3 Using AI to map skills and jobs

Ketamo et al. [38] argue that it is important for curriculum designers to stay up-to-date with the keywords being used in the industry that are valuable for *learners* when seeking jobs. They adopted AI, big data, and natural language processing to build a real-time tool for mapping the skills, competencies, and knowledge for both the job seekers and employers to keep them updated with the future job demands and forecast the competence needs.

The traditional curricula in higher education institutes constitutes of the expertise of the professors, lecturers, and domain-specific experts [39]. However, it is important to involve industry expertise in designing curricula. Thus higher education institutes should respond to the changing needs of today's industry. Therefore, AI can help investigate updated industry-relevant keywords and map them with skills and competencies.

## 3 Methodological framework

**Fig. 2:**
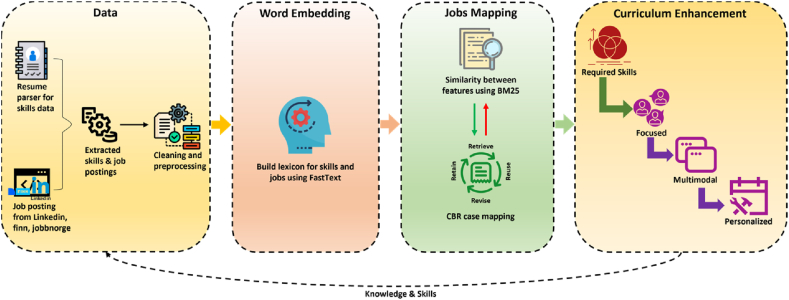
The framework of our proposed model consists of four layers: data, word embedding, jobs mapping, and curriculum enhancement layer.

This section discusses the details of our proposed study's techniques that analyze the Norwegian IT market's needs for required IT skills. We adopt a semantic-based approach to develop a cost-effective conceptual and analytical model to support students' study through market-required skills planning and to motivate them through career planning, thus improving their satisfaction and future employability by enhancing their computing curriculum. As illustrated in Fig. 2, our model consists of four modules: data, word embedding, job mapping, and curriculum enhancement layers. Finally, we present a case study analyzing the requirements of the future Norwegian technology market to enhance the curriculum of computing students in accordance with industrial demands.

### 3.1 Data layer

Data gathering from several sources is crucial for the first phase of our proposed framework. The data is gathered about careers and skills related to IT jobs posted on various platforms.

The data layer consists of several components where we interact with careers and skills components through state-of-the-art ML-based algorithms. It starts with resume collection as input and the jobs posted on the online platforms.

**Fig. 3:**
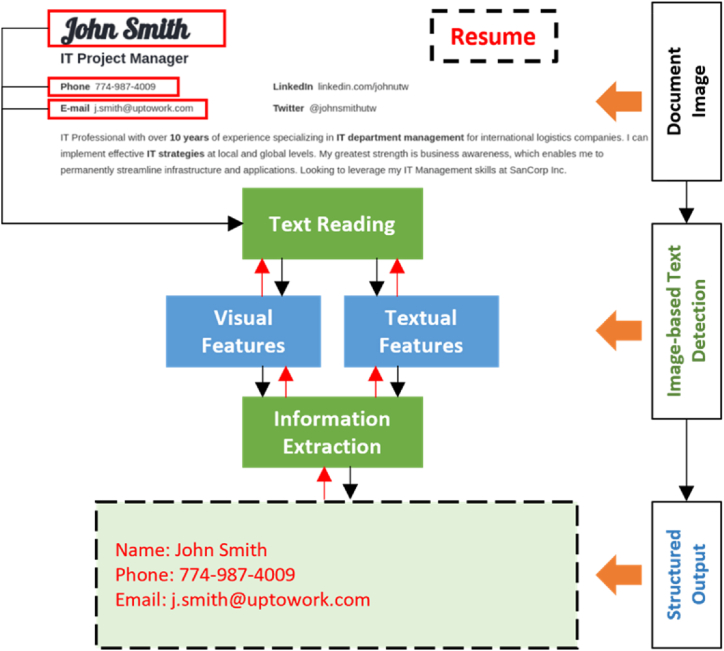
Image-based text detection from the resumes of the candidates is performed through an end-to-end text reading and information extraction framework [21].

We extract the skills and jobs by employing image-based text detection [21]. We use ResNet50 [40] as backbone of text detection module. To this end, we take input (resume) image *x*, and text detection branch *I* ∈ *R*^*h*×*w*×*d*^ where *h* is the height, *w* is the width, and *d* is the channel number of text detection branch *I*. The network predicts the text regions and extracts the data entities in three phases. First, it reads the text and detects the text using LSTM [41]. Second, a multimodal context block uses visual and textual information to extract resume's information. Third, an information extraction module finally extracts the contextual information from the candidates' resumes. Fig. 3 shows the architecture of image-based resume text detection used in this study.

(1) $$\bar{c_{i}}=\alpha \cdot Linear\left(\frac{1}{h^{\prime }w^{\prime }}\sum_{j}^{h^{\prime }w^{\prime }}c_{ij}\right)+\beta \cdot Linear\left(\tilde{c_{i}}\right)$$

Eq. (1) is the final formula for the information extraction from the resumes where $\bar{c_{i}}$ is the final context vector which is calculated by the weighted sum of visual and textual context information. *α* and *β* are the learnable weights, and *Linear* is the fully-connected layer.

In order to extract employment-related data, we investigated numerous job portals, such as *LinkedIn*^1^ and *finn.no*.^2^ *LinkedIn* is an online platform that enables job seekers to post their resumes and job firms to post jobs worldwide. *finn.no* is the leading job portal connecting job searchers with potential employers in Norway. Hundreds of job openings are added daily to this platform by top firms.

To extract the information for the job listings on the above-mentioned platforms, we input the structural information to sequence taggers for performing the Named Entity Recognition, which is necessary to find job-relevant keywords.

In the cleansing stage, we extracted only the relevant information required for the jobs by segmenting the related data and removing the irrelevant information such as Motivation, Job Location, etc. The model is trained on 1978 resumes [21] and tested on 497 resumes. We built a comprehensive dictionary on several aspects of candidates, e.g., programming languages, frameworks, technical skills, soft skills, certifications, etc. After collecting all the relevant data, we used R-tool to perform the final cleansing.

### 3.2 Embedding layer

In this layer, we use the FastText [22], an open source framework developed by Facebook, for efficient text classification of learned text representations and classifiers. This module's primary focus is to use job listings and skills data to perform the cleansing. In the data layer, we conducted data preprocessing, while the embedding layer performed additional data preprocessing to generate an IT domain skills map.

**Fig. 4:**
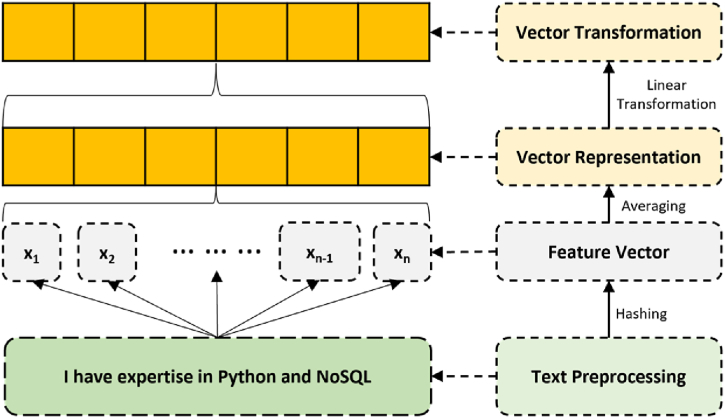
Architecture of FastText [22] embedding.

FastText [22] is a highly recommended algorithm in the words embedding domain. We use Eq. (2) to match the jobs and skills data from the *N* documents where *x*_*n*_ is the bag of vectors as shown in Fig. 4. *A* and *B* are the matrices, *ℓ* represents the softmax loss while *y*_*n*_ is the label of *n*-th document.

(2) $$\sum_{n=1}^{N}l\left(y_{n},BAx_{n}\right)$$

We adopted this specific algorithm due to its diverse nature of power distribution across the network. Our proposed model can encounter several misspelled jargon, e.g., *“pls”*, *“plz”*, and *“bcz”,* etc. So to avoid these misspellings and establish a shared origin of language linkages, we used this framework that also focuses on character-level embedding. A sampling chart eliminates common terms using FastText. This structure also reduces the amount of content in commonly used terms. Eq. (3) is used to calculate the probability *P* of a word using a frequency word:

(3) $$P\left(w\right)=\sqrt{\frac{t}{f\left(w\right)}}+\frac{t}{f\left(w\right)},f\left(w\right)=\frac{sum\left(w\right)}{T}$$

where *t* = 10*e*^−4^, *f*(*w*) is the frequency of words *w* occurrence and *T* is the total number of tokens. During the training phase, a term is eliminated if the probability of elimination is greater than a random selection from a uniform distribution between 0 and 1.

### 3.3 Mapping layer

We applied case-based reasoning to map the IT jobs with those of relevant skills. It is essential to analyze the samples generated by several systems, and to perform such an analysis, BM25 [42] is the outperforming matching algorithm. This algorithm is used to match learned skills with the skills in demand. This section further explains the CBR and BM25 in details.

#### 3.3.1 Case-based reasoning

Case-based reasoning (CBR) is a method of problem-solving that draws on prior responses to a same issue. It makes the assumption that knowledge can be obtained from past experience, that this aids in avoiding failure-prone routes, and that an existing solution may be modified to the present problem.

CBR may be found in a variety of settings. For instance, Google Maps uses it to calculate how long it will take you to go from point *A* to point *B* by analyzing the travel patterns of prior users. Even if your trip begins at two somewhat dissimilar locations, it generates conclusions about how long it will take. The premise behind CBR's “intelligent” use of information from previously resolved issues (or cases) is that the more similar two problems are, the more similar the solutions will be. The CBR technique typically entails looping through the following steps.

• Retrieval – retrieve the most relevant matching event from the database to the current event.
• Reuse – propose a new solution based on previous experiences and modify it according to the current needs.
• Revision – revise the solution's significance and importance in the new situation.
• Retain – keep the new approach for problem-solving in the memory system.

When mapping different examples against the same answer, CBR determines a similarity score by utilizing weighted averages to gauge how similar the cases are to one another. Eq. (4) is used to calculate the weighted average between the cases:

(4) $$S\left(N_{c},O_{c}\right)=\frac{1}{W}\sum_{i,j=1}^{n}SS\left(f_{i},f_{j}\right)\times W_{i}$$

where *S* is similarity between the new case *N*_*c*_ and old case *O*_*c*_, *SS* is the similarity score, and *W* = *W*_1_ + *W*_2_ + *W*_3_ + ···*W*_*n*_.

#### 3.3.2 BM25

We used the cutting-edge matching method BM25 [42] to determine how comparable the various characteristics were in various scenarios. According to an analysis of a sample of the weighted scores generated by multiple systems, the BM25 is the most successful at linking job skill terms from the prior case to job abilities in the current case. The mapping of any new CBR issue to an extant situation is an essential consideration. As a result, we matched the current example to the new issue using BM25. Eq. (5) displays the BM25 formula as follows:

(5) $$BM25\left(Q,L\right)=\sum_{t\in q}\;\left\{D_{l}F\left(q_{i}\right)\times \frac{\left(k_{1}+1\right)f\left(q_{i},D\right)}{k_{1}\left(\left(1-b\right)+b\times \left(\frac{l_{L}}{l_{av}}\right)\right)+f\left(q_{i},D\right)}\right\}$$

where *BM* stands for best-match, *k*_1_ and *b* are the constants used for bestmatching, *L* represents the new case, and *Q* represents the existing case, *D*_*l*_ is the document length while *F* is the frequency of occurrences.

### 3.4 Curriculum enhancement layer

The curriculum should be updated based on the following factors. (1) *maximize coverage:* it should be able to address the deficiencies of as many students as it can regarding their skills and knowledge; (ii) *feasibility:* a limited number of teachers should be taken into consideration; and (iii) *inclusive:* the needs of students with diverse background should also be considered [24]. These factors pave the basis for our curriculum enhancement module.

After mapping the skills with jobs, we get the required skills in the industry. Based on the requirements, we use a feedback loop proposed by Wensheng Wu [24] to update the curriculum based on the feedback of our mapping layer. The revision of the curriculum is done in three stages.

1. *Focused:* The primary focus of revision should be to maximize the coverage, and it should focus on the key skills and knowledge sought in the industry.
2. *Multimodal:* The revision should come in a way that a minimum number of staffing is required, and the updated skills training can be offered as elective courses, recommending online classes, revising the existing courses, developing the new courses, and creating the mini-courses for that purpose.
3. *Personalized:* It is recommended to offer a selection of pre-requisites for the new courses so that the students who lack certain skills can take some of *multimodal* type courses to develop those skills and knowledge for additional training.

After making the mandatory revisions and updates to the curriculum, the data layer can again be updated with newly produced knowledge and skills.

## 4 Case study of Norwegian job market in data science and software engineering

Using data from *LinkedIn.com* and *finn.no*, this section presents a case study of the Norwegian IT employment market in the disciplines of data science, artificial intelligence, and software engineering. On June 30, 2022, we listed over 300 open positions for big data developers and engineers, as well as database systema administrators. Also, we gathered the resumes of more than 50 students from Norwegian institutions to be used as information about their education and training. We utilized our suggested technique to pinpoint the trends in the Norwegian labor market for the chosen industries as well as the relevant qualifications needed for that particular professions. Via the mapping layer, we linked the positions with the talents, and the insights about the patterns are given in this area.

### 4.1 IT jobs market in Norway

**Fig. 5:**
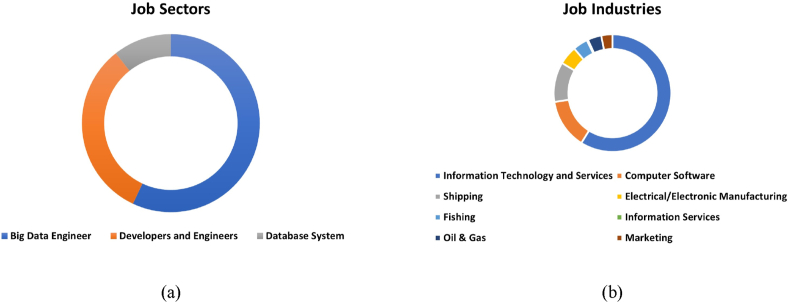
Norwegian IT job market across several areas of life.

**Fig. 6:**
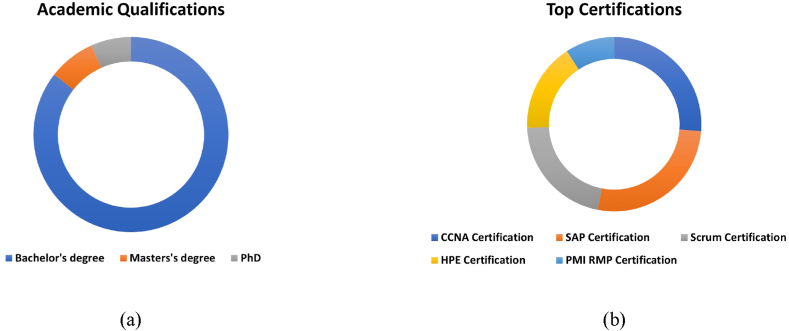
Academic qualifications and certifications required in the Norwegian jobs market.

We present the employment trends for big data developers and engineers as well as database system administrators in Norway's technology sector. Fig. 5 shows the Norwegian IT jobs market where it can be seen in Fig. 5a that Big Data Engineers dominate over 50% jobs, and the main industries are information technology and service providers (see Fig. 5b). From Fig. 6a it is visible that Bachelor's is the main requirement for most IT jobs while surprisingly, Master's and Ph.D. are also widely sought requirements for the IT jobs in Norway. Among the top required certifications, CCNA is the most sought certification as shown in Fig. 6b.

**Fig. 7:**
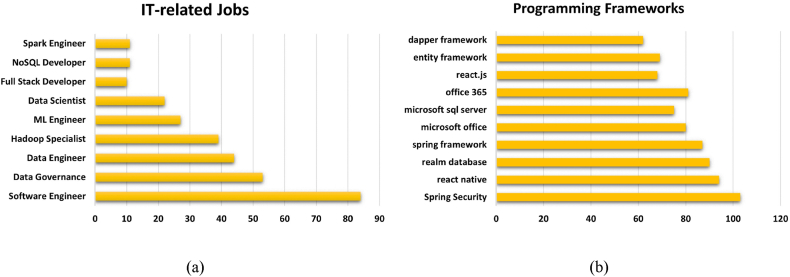
Norwegian IT-related jobs and recommended programming frameworks.

Furthermore, we show the IT-related jobs and programming frameworks required by the Norwegian industries in Fig. 7. It can be seen that Software Engineering dominates the other fields (see Fig. 7a). The *Spring Security* framework is the top requirement for Software Engineer jobs as shown in Fig. 7b. These recommendations are useful for the institutes to develop their curriculum according to the industrial needs and future planning of their curriculum policies. Our proposed framework extracts the skills and jobs from the resumes and online platform and maps them using the mapping layer. In the following sections, we also show the recommendations for data science, software engineering, and NoSQL jobs in the Norwegian IT market.

### 4.2 Recommendations for data science jobs

**Fig. 8:**
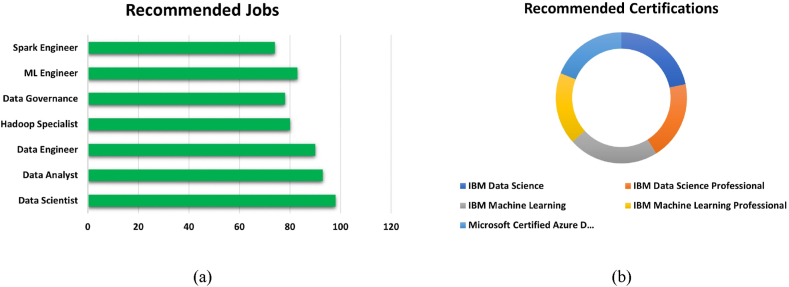
Recommended jobs and certifications in the field of data science.

The recommended jobs and certifications required for those jobs in the data science field are shown in Fig. 8. The curriculum enhancement module will educate the learners and teachers in accordance with the prescribed job titles and technical skills required for these positions. It can be seen that *Data Scientist* is the mainly sought job among other data related jobs as illustrated in Fig. 8a. Accordingly, the recommended certification from our mapping layer are IBM Data Science and IBM Data Science Professional, while IBM Machine Learning Professional is also now one of the widely required certification in data science domain (see Fig. 8b).

**Fig. 9:**
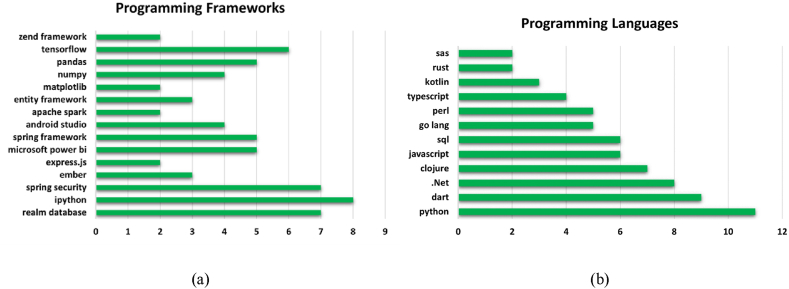
Recommended programming frameworks and languages in the field of data science.

Among the top programming frameworks for data science, *iPython* is the top of the list in Fig. 9a, while *Python* is one of the top programming languages sought by the industries as shown in Fig. 9b. Hence, computing learning enthusiasts should focus on learning the relevant frameworks and language if they want to pursue their careers in the data science field.

### 4.3 Recommendations for software engineering jobs

**Fig. 10:**
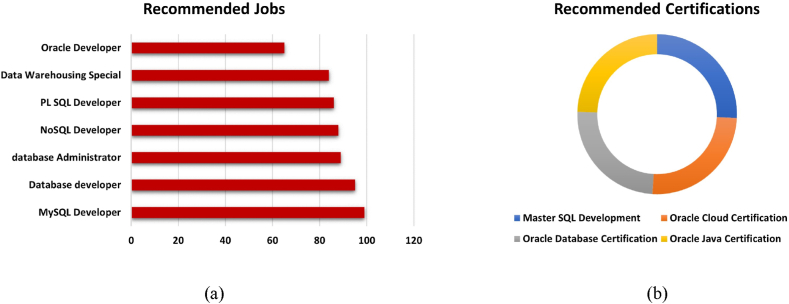
Recommended jobs and certifications in the field of software engineering.

**Fig. 11:**
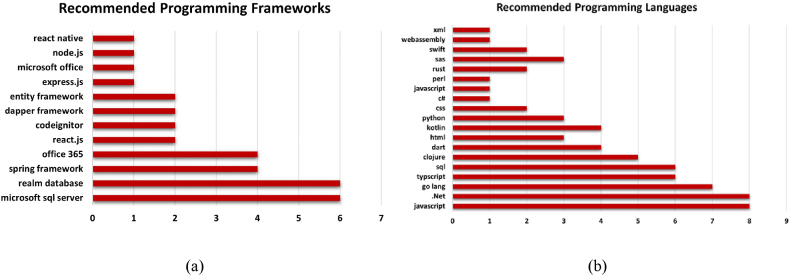
Recommended programming frameworks and languages in the field of software engineering.

We show the most relevant skills, certifications, and qualifications required for the software engineering jobs in this section. The analysis shows that MySQL developers are widely sought across software engineering disciplines, while mastering SQL development is also mandatory for those professionals as shown in Fig. 10a and b. While among the recommended programming frameworks, *Microsoft SQL Server* and *realme* database are top of the list as mentioned in Fig. 11a. Similarly, *go lang* and *javascript* stay on top of the list, which is widely sought in the IT industry for software engineering-related jobs (see Fig. 11b). So, the course planners of software engineering should focus on the recommendations to update the course curriculum while enhancing the employability of students in the industry.

### 4.4 Recommendations for SQL jobs

**Fig. 12:**
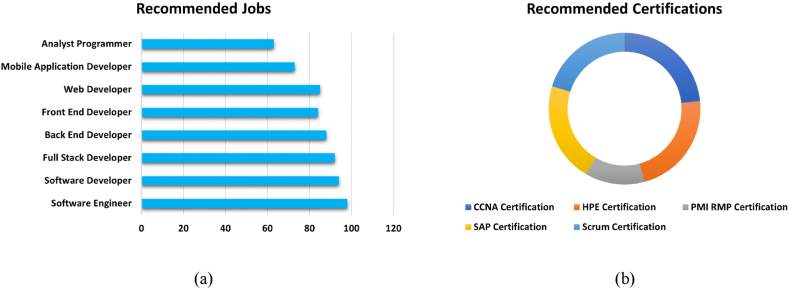
Recommended jobs and certifications in the field of SQL.

**Fig. 13:**
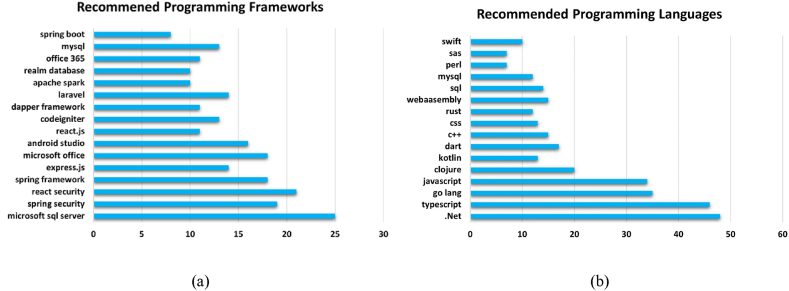
Recommended programming frameworks and languages in the field of SQL.

We also analyzed SQL-related jobs in the industry, which are closely related to software engineering. It is clearly seen that for SQL-related jobs, *Software Engineers* are widely sought while CCNA certification stays on top of the list (see Fig. 12a and b). From Fig. 13, it is visible that *MySQL* was least sought programming frameworks in June 2022 while *.Net* makes top of the list for programming languages. It is necessary to mention here that *.Net* is itself a framework, and mostly *Java/C#* are used in the back-end of this framework.

So, *Jave/C#* are the widely used programming languages for SQL-related jobs. Thus, the learners should focus on developing their skills in *Java/C#* and *Microsoft SQL Server* development. The teachers should also revise the curriculum accordingly.

## 5 Discussions

This section discusses the impact of our proposed framework on the case study's outcomes. We take the students' resumes and jobs posted on online jobs platforms as input in our data layer, which preprocesses the data and take it into the words embedding layer, where we build a lexicon about the words. Afterward, we apply the jobs mapping layer to the words obtained through the words embedding layer, map the keywords using the BM25 matching algorithm, and apply CBR to match skills with jobs. Finally, we apply a curriculum enhancement layer that, based on the acquired skills, proposes a strategy to build market-required skills into students' curricula delivered at varsities.

The recommendations show that Bachelor's is the essential criterion for entering the IT jobs market in Norway (see Fig. 6a). However, for some specific tasks, the companies now require specialists having completed their master's and PhD's in some cases. The IT job market is growing in Norway as well as worldwide. Start-ups and mature companies need specific skills for their operations; therefore, the students need to obtain those skills in their Bachelor's programs. Additionally, the industry needs more software and data engineers in its operations. Hence, the curriculum can be updated to deliver more software and data skills rather than developing general computing skills by using the focused strategy in the curriculum enhancement layer. The multimodal technique can be used to deliver knowledge about the most required certifications in the market such as those available in Fig. 6, Fig. 8, Fig. 10, Fig. 12b. The students can also register themselves for the industry-relevant certifications on online platforms, e.g., *edX*,^3^ *Coursera*,^4^ *Udemy*,^5^ etc.

Programming languages are one of the essential skills that computing graduates must learn during their course of studies. Employers also focus on programming languages while conducting interviews with the candidates. A computing graduate must learn at least one programming language before graduating and entering the industry. From the recommendations, it can be seen that data science industry requires *Python* the most while software engineering related jobs require students to have a significant aptitude in *javascript*, *.Net*, and *typescript* (see Fig. 9, Fig. 11, Fig. 13b). If the faculty does not qualify to teach either of the most required programming languages, then a suitable curriculum must be developed by hiring some industry practitioners or letting students acquire those language skills via online platforms. Similarly, the programming frameworks are a tool that provides the ready-made components for software development, and these components help speed up the development process. From Fig. 9, Fig. 11, Fig. 13a, it can be seen that *ipython*, *Realme Database*, and *Microsoft SQL Server* are most recommended programming frameworks for data science, software engineering, and SQL jobs, relatively.

The skills mentioned in the resumes are the criteria by which an employer will judge the candidates, and having those skills that one specific industry requires to perform its operations outstands a candidate, among others. Therefore, it is imperative to learn industry-relevant skills if someone is interested in pursuing his/her career in the industry after graduation or wants to become an entrepreneur. By leveraging our proposed deep learning and big data-based framework, the industry and academia can benefit from training the students in the skills required by the industries.

Norway's sustainable policies focus on developing digital transformation strategies in its industrial sector, and for that purpose, hundreds of IT jobs are posted on online platforms in several industries. Many national and international candidates are being hired to meet the requirements of the industrial workforce. However, there is still a vast gap between the industry's labor demand and the workforce being trained at varsities. There is a huge need to fill this gap by developing an industry-relevant skills-based workforce that can readily work on the industry's required tasks. By using our proposed framework, we argue that students will easily be able to be accepted into the industry after completing their graduation.

This study contributes to Computing Education Research (CER) by providing a paradigm that links the skills of young Norwegians in higher education with the technology job market. A data-driven gap study shows the skills gap between the Norwegian IT job market and computer science pupils. We believe this is Norway's first such report. The study's results add to the literature by offering a complete paradigm that ties job market skills to computer science students' skills in Norway. This knowledge can help course managers spot curricular gaps and create job market-relevant classes. This study also helps students build job paths and improve interest and recall.

The proposed framework has a number of advantages over current research. Initially, the research utilizes large employment data from online job portals to determine the skills required by the Norwegian IT job market. Second, the study investigates the most recent developments in the skills required to promote the social impact of learning. Thirdly, the model utilizes case-based reasoning to map the skills disparity between market-required skills and student-acquired skills. To improve the content of the computing curriculum, the study proposes a curriculum enhancement strategy based on the requisite skills of the labor market.

Our proposed framework can be used for any subject by training the models for other areas, e.g., business, natural sciences, engineering sciences, etc. The framework needs the data, and recommendations about other industries can also be made that can help students better prepare for industrial jobs. A running product in the future will help both academia and industry to benefit from our services. For example, varsities can update their curriculum for core subjects based on our recommendations. Industries can post their required skills in advance to help the students acquire those skills in advance.

## 6 Threats to validity

Since the case study was performed on the data available online and the data provided by the candidates from the Norwegian varsities. The threats to the validity of our proposed study are the following. We used 50 resumes from the candidates and online data available on job posting platforms. We used the data analytics method to perform an analysis of the data. There are external threats to validity, such as a lack of expert evaluation in the context of mapping required skills. There can be ignorance in using the relevant factors for evaluating experimental performance. The subject selection can also be a factor of external threats to the validity of this proposed research.

## 7 Conclusions and future directions

We have presented a case study on the Norwegian IT marker-required skills and analytical insights into the industry-relevant skills. Mostly, employers are in search of skilled workers who can efficiently deliver their best to meet the project's deliverable requirements. Therefore, it has become crucial to develop such courses or enhance the curricula that can develop job-relevant skills and knowledge among the students. The learners, teachers, and researchers are the main actors in designing curricula. We, in this work, mainly focused on developing IT-related skills and enhancing the computing curriculum according to the industrial demands.

We leverage our quad-layered architecture consisting of data, an embedding, a mapping, and curriculum enhancement layers to make recommendation for curriculum enhancement. In the data layer, we input the data from candidates’ resumes and online posted jobs; after performing an initial screening and preprocessing, we send this data to the embedding layer, where by using the FastText, we perform character-level embedding on the keywords. After this, we use CBR and BM25 to match the skills with the requirements, and the final curriculum enhancement layer is updated with the required skills. In the final stage, we update the computing course curriculum based on the recommendations. We performed the analysis using our proposed module for Data Science and Software Engineering courses and jobs.

In the future, we will employ the proposed strategy in other computing courses and recommend enhancing other courses’ curricula. We will also investigate the effective training methods that enable computing graduates to develop industry and academic relevant hard and soft skills. We will try to learn from the experiences of internees, experts, and industry practitioners.

## Author contribution statement

Muhammad Umair Hassan: Conceived and designed the experiments; Performed the experiments; Analyzed and interpreted the data; Contributed reagents, materials, analysis tools or data; Wrote the paper.

Saleh Alaliyat; Raheem Sarwar; Ibrahim A. Hameed: Analyzed and interpreted the data; Contributed reagents, materials, analysis tools or data.

Raheel Nawaz: Contributed reagents, materials, analysis tools or data.

## Data availability statement

The authors do not have permission to share data.

## Footnotes

1 https://www.linkedin.com/

2 https://www.finn.no/

3 https://www.edx.org/

4 https://www.coursera.org/

5 https://www.udemy.com/
